# Molecular and MALDI-TOF identification of ticks and tick-associated bacteria in Mali

**DOI:** 10.1371/journal.pntd.0005762

**Published:** 2017-07-24

**Authors:** Adama Zan Diarra, Lionel Almeras, Maureen Laroche, Jean-Michel Berenger, Abdoulaye K. Koné, Zakaria Bocoum, Abdoulaye Dabo, Ogobara Doumbo, Didier Raoult, Philippe Parola

**Affiliations:** 1 Aix Marseille Université, UM63, CNRS 7278, IRD 198, INSERM 1095, AP-HM, IHU - Méditerranée Infection, Marseille, France; 2 Malaria Research and Training Center, Département d’Epidémiologie des Affections Parasitaires, Faculté de Médecine et d’Odontostomatologie, Faculté de Pharmacie, USTTB, Bamako, Mali; 3 Unité de Parasitologie et Entomologie, Département des Maladies Infectieuses, Institut de Recherche Biomédicale des Armées, Marseille, France; 4 Laboratoire Central Vétérinaire, Bamako, Mali; Medical College of Wisconsin, UNITED STATES

## Abstract

Ticks are considered the second vector of human and animal diseases after mosquitoes. Therefore, identification of ticks and associated pathogens is an important step in the management of these vectors. In recent years, Matrix-assisted laser desorption/ionization time-of-flight mass spectrometry (MALDI-TOF MS) has been reported as a promising method for the identification of arthropods including ticks. The objective of this study was to improve the conditions for the preparation of tick samples for their identification by MALDI-TOF MS from field-collected ethanol-stored Malian samples and to evaluate the capacity of this technology to distinguish infected and uninfected ticks. A total of 1,333 ticks were collected from mammals in three distinct sites from Mali. Morphological identification allowed classification of ticks into 6 species including *Amblyomma variegatum*, *Hyalomma truncatum*, *Hyalomma marginatum rufipes*, *Rhipicephalus (Boophilus) microplus*, *Rhipicephalus evertsi evertsi* and *Rhipicephalus sanguineus sl*. Among those, 471 ticks were randomly selected for molecular and proteomic analyses. Tick legs submitted to MALDI-TOF MS revealed a concordant morpho/molecular identification of 99.6%. The inclusion in our MALDI-TOF MS arthropod database of MS reference spectra from ethanol-preserved tick leg specimens was required to obtain reliable identification. When tested by molecular tools, 76.6%, 37.6%, 20.8% and 1.1% of the specimens tested were positive for *Rickettsia* spp., *Coxiella burnetii*, *Anaplasmataceae* and *Borrelia* spp., respectively. These results support the fact that MALDI-TOF is a reliable tool for the identification of ticks conserved in alcohol and enhances knowledge about the diversity of tick species and pathogens transmitted by ticks circulating in Mali.

## Author summary

Ticks are among the most important vectors and reservoirs of several animal and human pathogens such as viruses, bacteria and protozoa. However, very few studies have been done on ticks in Mali. At present, little information is available about tick species infesting livestock or human tick-borne diseases transmitted in Mali. The identification of tick species and the determination of pathogens associated are essential to evaluate epidemiology and risks of human and animal diseases: the One Health approach. Current identification methods are time consuming, expensive and laborious. Previous studies have shown that MALDI-TOF mass spectrometry analyses may allow accurate tick species identification. A recent study suggested that it was possible to identify ticks preserved in alcohol by MALDI-TOF MS. The aim of the present study was to improve tick leg sample preparation conditions for their identification by MALDI-TOF MS from Malian ethanol-preserved specimens collected in the field. This study provided 99.4% concordance between morphological and MALDI-TOF identification. The detection of microorganisms was also performed by molecular biology revealing the presence of the presence of *Rickettsia* spp., *Coxiella burnetii*, *Borrelia* spp. and *Anaplasmataceae*. These results support the use of MALDI-TOF MS in entomology, tick diseases epidemiology and improve the knowledge of tick species-diversity and tick-borne pathogens circulating in Mali.

## Introduction

Ticks are bloodsucking arthropods that parasitize most of the vertebrates in the world and occasionally bite humans [1]. About 900 tick species have been identified and classified worldwide [2]. In Africa, the number of tick species indexed is 223, including 180 hard and 43 soft ticks [2]. Currently, ticks are considered the second most important vector of human disease after mosquitoes and can transmit bacterial [1], viral [3] and protozoan pathogens [4]. A significant number of these pathogens are of exceptional importance, as they are responsible for high morbidity and mortality in humans and animals [1]. Identification of tick species is an important step in epidemiological studies, in order to establish tick species distribution maps and to characterize tick fauna and seasonal trends [5,6].

In Mali, a West African country, livestock farming is an essential economical factor. At present, there are few studies on tick species that infest cattle or tick-borne diseases transmitted in Mali. To date, 23 tick species belonging to six genera have been categorized in Mali [7–9]. Among them, *Amblyomma (Am*.*) variegatum*, *Rhipicephalus (Rh*.*)* spp. and *Hyalomma (Hy*.*)* spp. are the main ticks monitored by Malian veterinarians for their effects on livestock healthcare and productivity [10]. Other public health problems, such as tuberculosis, AIDS or malaria, take precedence over tick-borne diseases (TBDs), which are little explored by medical doctors.

Several bacteria were detected in ticks from Mali. Spotted fever group rickettsiae were detected, including *Rickettsia africae* in *Am*. *variegatum*, *R*. *aeschlimannii* in *Hy*. *marginatum rufipes*, and *R*. *massiliae in Rhipicephalus* spp., all three being human pathogens [11]. An *Ehrlichia* sp. of unknown pathogenicity, *Ehrlichia* Erm58, was detected in *Rh*. *mushamae* [11]. More recently, *Borrelia theileri*, the agent of bovine and equine borreliosis, and *B*. *crocidurae*, agents of relapsing fever in humans, have been detected in *Rh*. *geigyi* and *Ornithodoros sonrai*, respectively [12–14].

To study and control ticks and TBD transmission, accurate identification of tick species and determination of their infectious status are essential [1]. Currently, tick identification is principally conducted by observing morphological characteristics. However, it is limited by entomological expertise, dichotomous keys availability, tick integrity or engorged status [9]. Molecular tools have been used as an alternative to overcome the limitations of morphological identification [15]. Sequencing of several genes has been used, including ribosomal sub-units (e.g., *12S*, *16S* or *18S*), the cytochrome c oxidase unit I (*COI*), or the internal transcribed spacer [16]. These techniques are generally time-consuming, laborious and can be expensive, preventing their use in large scale studies [17–20]. Moreover, the absence of a consensus gene target sequence for tick identification and/or the comprehensiveness of genomic databases are additional factors hampering their use [16].

Recently an alternative tool based on the analysis of protein profiles resulting from matrix-assisted laser desorption/ionization time-of-flight mass spectrometry (MALDI-TOF MS) analysis has been explored to identify arthropods [21]. MALDI-TOF MS has been used to identify tick species [22–24] and to determine tick infectious status [25–27].

However, tick collection usually takes place far from analytical laboratories and therefore requires proper storage of samples. Ticks are generally stored either alive, at -20°C, or in alcohol. Although alcohol storage is cheaper and easier, especially in African countries, previous studies reported that the use of fresh (i.e., recently dead) or frozen specimens led to more reproducible and better MS spectra compared to the alcohol preservation mode for ticks [24] [28], and also for other arthropod families[29,30]. In a recent study, it was demonstrated that long-term tick storage in alcohol altered MS profiles, which did not provide conclusive identification following in-house MS reference spectra database-querying containing MS spectra from counterpart fresh tick species. Nevertheless, the upgrading of the in-house MS reference spectra database of specimens stored in alcohol allowed correct identification of ticks at the species level, also underlining the reproducibility and specificity of MS profiles for tick specimens stored in alcohol [31].

The goal of the present work was to determine tick population diversity and associated pathogens from alcohol stored specimens collected on cattle in Mali by using MALDI-TOF MS and molecular approaches with specimens collected in the field. First, optimized sample preparation conditions for ticks stored in ethanol for MALDI-TOF MS analysis were established. Second, based on morphological and molecular identification of ticks, an MS reference spectra database was created and tested blindly using new tick specimens. In addition, tick-associated bacterial pathogens were screened by molecular biology on half-tick body parts and leg MS spectra from ticks mono-infected or not by bacterial pathogenic agents, and they were compared to assess the efficiency of this proteomic tool for classification of ticks according to their infectious status.

## Materials and methods

### Ethical considerations

Tick collection protocols were developed as of a large study under the GIRAFE programme, UMI 3189 and MSHP-MRTC HFV project. The protocols were cleared by the FMPOS IRB in 2015 and 2016. Verbal informed consent was obtained from managers of the livestock selected for tick sampling directly on mammals. The collection of ticks on domestic animals did not involve national parks or other protected areas or endangered or protected species.

### Study sites and collection period

**Fig 1:**
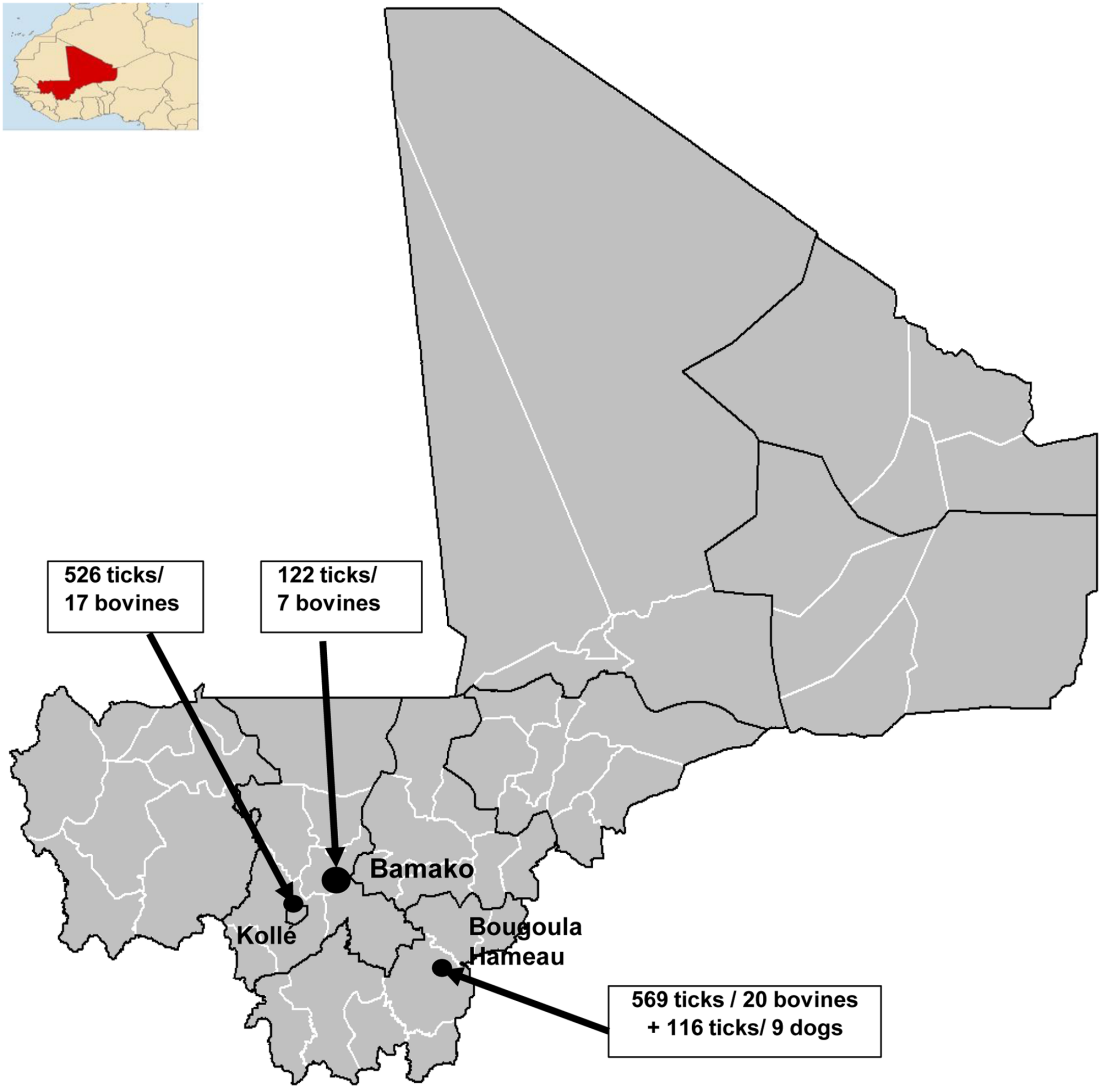
Map of Mali showing the sites where the ticks were collected for our study and number of ticks collected and number of cattle prospected per site.

Ticks were collected from three localities in Mali, including Bamako, Kollé and Bougoula Hameau, in September 2015 and August 2016 (Fig 1). Bamako, the capital city of Mali, is an urban area surrounded by hills. The climate is Sahelian-type with two distinct seasons, the dry season (i.e., from November to May) and the rainy season (i.e., from June to October). The total amount of precipitation was less than 900 milliliters in 2009. Kollé is a rural village located about 60 km southwest of the capital. Agriculture, livestock farming and small businesses are the main economic activities of the village. The village, located on a flat land with submersible and dry areas, presents a Sahelian-type climate with two distinct seasons, a rainy (i.e., from June to November with maximum rainfall in August-September of 350 to 400 milliliters) and a dry season (i.e., from December to May with a cool period in December- February and a warm period in March-May). The third site was Bougoula Hameau, a suburban village, located at 4 km of Sikasso town and it was situated at 374 km southeast of Bamako by road. The climate is of Sudanese type, under the influence of the humid forest with a rainy (i.e., from May to October) and a dry season (i.e., from November to April). The annual rainfall can vary from 1,200 to 1,800 milliliters, depending on the year. These climatic conditions are appropriate for agricultural and livestock farming.

### Collection method

Ticks were collected from domestic animals and cattle. Examination of all body parts was conducted from the tail to the head of the animal to detect ticks on the skin. All ticks (engorged and non-engorged) were collected manually with forceps. The ticks of the same animal were counted, pooled in the same tube and stored at room temperature in 70% v/v ethanol (ticks collected in September 2015) or frozen at -20°C (ticks collected in August 2016) until morphological, molecular and MALDI-TOF MS analyses. Ticks were transferred from MRTC (Bamako, Mali) to the URMITE laboratories (Marseille, France) for analysis.

### Morphological identification

Ticks were identified morphologically to the species level firstly by a PhD student and then checked by expert tick entomologists using previously established taxonomic identification keys [9]. Tick identification and gender determination were performed under microscope at a magnification of ×56 (Zeiss Axio Zoom.V16, Zeiss, Marly le Roi, France). The tick genera, species, gender, host and animal number, collection site and date were codified to include this information on the tube.

### Ticks dissection and sample preparation

Each tick was dissected with a new sterile surgical blade to remove the legs, which were used for MALDI-TOF MS analyses. The rest of the tick was longitudinally cut in two equal parts. The half part with legs cut off was immediately used for molecular biology, and the second half was stored frozen as a backup sample for any additional analysis.

### DNA extraction

Each half-tick without legs was transferred to a 1.5 mL tube containing 180 μL of G2 lysis buffer and 20 μL proteinase K (Qiagen, Hilden, Germany), and incubated at 56°C overnight. DNA extraction from the half-tick was performed with an EZ1 DNA Tissue Kit (Qiagen) according to manufacturer recommendations. The DNA from each sample was eluted with 100 μL of Tris-EDTA (TE) buffer (Qiagen) and was either immediately used or stored at -20°C until use.

### Molecular identification of ticks

Standard PCR, using an automated DNA thermal cycler amplifying a 405-base pair fragment of the mitochondrial 12S RNA gene (Table 1), was used for tick identification to the species level, as described previously [31]. The 16S RNA gene was used to confirm all *Rhipicepalus (Boophilus) microplus* identification. DNA from *Am*. *variegatum* specimens reared at the laboratory was used as positive control. PCR products of the positive samples were purified and sequenced as described previously [31]. The sequences were assembled and analyzed using the ChromasPro software (version 1.34) (Technelysium Pty. Ltd., Tewantin, Australia), and were then blasted against GenBank (http://blast.ncbi.nlm.nih.gov).

**Table 1:** Primers and probes used for real-time quantitative and standard PCR in this study.

Microorganisms	Targeted sequence	Primers f, r (5’-3’) and probes p (6FAM-TAMRA)	References
**qPCR primers**			
***Rickettsia spp***.	*gltA(RKND03)*	f_GTGAATGAAAGATTACACTATTTAT	[72]
		r_GTATCTTAGCAATCATTCTAATAGC	
		p_CTATTATGCTTGCGGCTGTCGGTTC	
***R***. ***africae***	*poT15-dam2*	f_TGCAACACGAAGCACAAAAC	[32]
		r_CCTCTTGCGAAACTCTACTT	
		p_TGACGTGTGGATTCGAGCACCGGA	
***Anaplasma spp***.	*23SrRNA (TtAna)*	f_TGACAGCGTACCTTTTGCAT	[34]
		r_TGGAGGACCGAACCTGTTAC	
		p_GGATTAGACCCGAAACCAAG	
***Borrelia spp***.	*(Bor ITS4)*	f_GGCTTCGGGTCTACCACATCTA	[62]
		r_CCGGGAGGGGAGTGAAATAG	
		p_TGCAAAAGGCACGCCATCACC	
	*(Bor_16S)*	f_AGCCTTTAAAGCTTCGCTTGTAG	[73]
		r_GCCTCCCGTAGGAGTCTGG	
		p_CCGGCCTGAGAGGGTGAACGG	
***C***. ***burnetii***	*(IS30A)*	f_CGCTGACCTACAGAAATATGTCC	[74]
		r_GGGGTAAGTAAATAATACCTTCTGG	
		p_CATGAAGCGATTTATCAATACGTGTATG	
***Bartonella spp***.	*(Barto ITS2)*	f_GATGCCGGGGAAGGTTTTC	[75]
		r_GCCTGGGAGGACTTGAACCT	
		p_GCGCGCGCTTGATAAGCGTG	
**Standard PCR primers**			
***Rickettsia spp***.	*gltA*	f_ATGACCAATGAAAATAATAAT	[33]
		r_CTTATACTCTCTATGTACA	
***Anaplasma spp***.	*23SrRNA*	f_ATAAGCTGCGGGGAATTGT	[34]
		r_TGCAAAAGGTACGCTGTCAC	
***Borrelia spp***.	*flaB*	f_TGGTATGGGAGTTTCTGG	[35]
		r_ TAAGCTGACTAATACTAATTACCC	

### Detection of pathogens

Quantitative PCR was performed according to the manufacturer's protocol using a PCR detection system; a CFX Connect^™^ Real-Time (Bio-Rad) with the Eurogentec Takyon qPCR kit (Takyon, Eurogentec, Belgium). The qPCR reaction contained 10 μl of Takyon Master Mix (Takyon, Eurogentec, Belgium), 3.5 μl sterile distilled water, 0.5 μl of each of the primers and probe and 5 μl of the DNA extract. A total of 471 samples were screened using primers and probes, targeting specific sequences of the following bacterial pathogens: *Rickettsia* spp., *Anaplasmataceae* spp., *Borrelia* spp., *Bartonella* spp. and *Coxiella burnetii* (Table 1). For *Borrelia* spp we used 2 genes, the 16S *Borrelia* gene first and all the ticks that were positive for this gene were retested by ITS4 for confirmation. Only samples positive for both genes (16S borrelia and ITS4) were considered positive. Positive samples for *Rickettsia* spp. were then submitted to a qPCR system specific for detecting *R*. *africae* [32]. Negative samples for *R*. *africae* but positive for *Rickettsia* spp. were submitted to *gltA* gene sequencing to determine *Rickettsia* species [33]. All ticks positive either for *Anaplasmataceae* spp. were submitted to amplification using standard PCR and sequencing to identify the bacteria species [34,35]. Ticks that were positive for Borrelia spp for both the 16S Borrelia gene and ITS4 were submitted to amplification using standard PCR and sequencing [33]. PCR tests were considered positive when the cycle threshold (Ct) was lower than 36 [36]. The DNA from *Rickettsia montanensis*, *Bartonella elizabethae*, *Anaplasma phagocytophilum*, *Coxiella burnetii* and *Borrelia crocidurae* was used as positive controls and mix as negative controls in PCR, respectively. All these bacteria come from the strains of culture of our laboratory and *Borrelia crocidurae was* cultured in Barbour-Stoenner-Kelly (BSK-H) liquid medium supplemented with rabbit serum. Only samples considered as negative (i.e., Ct ≥ 36 for all bacteria tested), were submitted to 12 S tick gene amplification to control the correctness of DNA extraction.

### MALDI-TOF MS analyses

#### Optimization of tick sample preparation prior to submission for MALDI-TOF MS

**Fig 2:**
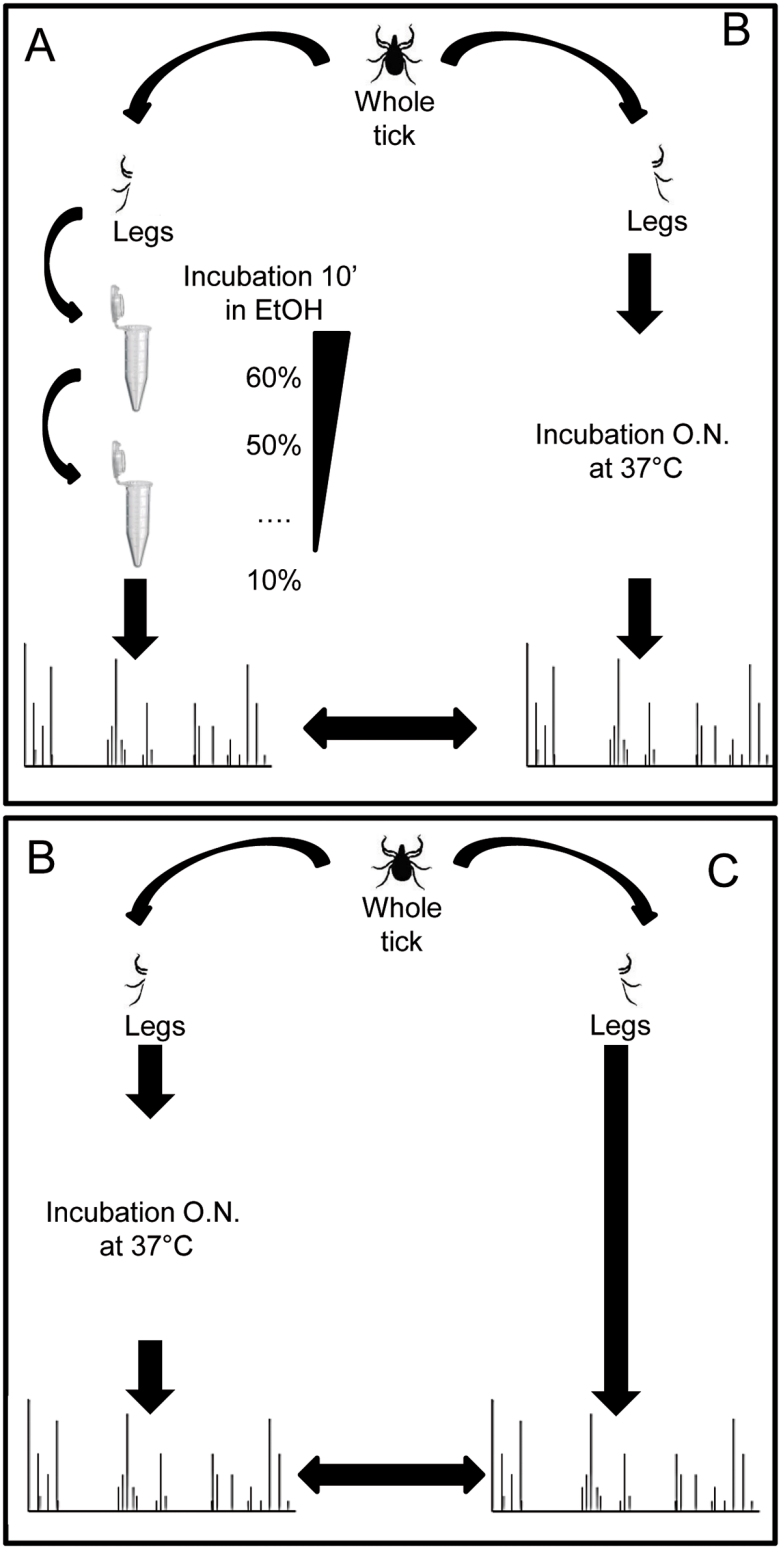
Protocol design of ticks treatment for MALDI- TOF MS analyses. “De-alcoholization” (A), “dry” (B) and “direct” (C) protocols for sample preparation were illustrated.

Three protocols were tested for tick leg sample preparation from specimens stored in 70% ethanol to determine the one exhibiting the best MS results (Fig 2). For each comparison, four legs from one side were cut off to test the first protocol, and the four other legs from the same tick were used to assess the second protocol. The criteria for protocol selection were by order of importance, intra-species MS spectra reproducibility and the simplicity of the protocol. The reference protocol, called “de-alcoholization” consisted of a 10-minute successive incubation washing of the whole tick (i.e., prior to dissection) in decreasing ethanol concentrations from 70% to 10% (v/v). A final wash in distilled water was conducted prior to sample drying on sterile filter paper and tick leg cutting as previously described [31]. The second protocol, called “direct-MS,” consisted in drying the whole tick on filter paper, and the cutting of the four legs, which were directly homogenized for MALDI-TOF analysis. The third protocol, called “dry-MS”, was similar to the “direct-MS” protocol, except those cuts off legs were dried overnight at 37°C prior to homogenization. Whatever the protocol used, tick legs were homogenized with the same method. A pinch of glass powder (Sigma, Lyon, France) was added to the tick legs, plus 40 μL of a mix of 70% (v/v) formic acid and 50% (v/v) acetonitrile (Fluka, Buchs, Switzerland). The legs were then homogenized using the TissueLyser apparatus (QIAGEN, Germany) with the following setup parameters as previously described [28]. First, the “de-alcoholization” protocol was compared to the “dry-MS” one, and then the “dry-MS” protocol was compared to the “direct-MS” protocol. The selected protocol was then applied to the other tick legs homogenized in the present study. The tick legs stored frozen were directly homogenized and used for MALDI-TOF MS.

### Sample loading on the target plate

The homogenized tick legs were centrifuged at 2000 g for 30 seconds and 1 μL of the supernatant from each sample was carefully dropped onto the MALDI-TOF target plate as previously described [28]. Each spot was then recovered with 1 μL of CHCA matrix solution composed of saturated α-cyano-4-hydroxycynnamic acid (Sigma, Lyon. France), 50% acetonitrile (v/v), 2.5% trifluoroacetic acid (v/v) (Aldrich, Dorset, UK) and HPLC-grade water [16]. The target plate, after drying for several minutes at room temperature, was introduced into the Microflex LT MALDI-TOF Mass Spectrometer device (Bruker Daltonics, Germany) for analysis. The loading of the MS target plate, the matrix quality, and the performance of the MALDI-TOF were performed as previously described [28].

### MALDI-TOF MS parameters

Protein mass profiles were obtained using a Microflex LT MALDI-TOF mass spectrometer (Bruker Daltonics, Germany) using parameters previously described [31]. The spectrum profiles obtained were visualized with Flex analysis v.3.3 software and exported to ClinProTools software v.2.2 and MALDI-Biotyper v.3.0. (Bruker Daltonics, Germany) for analysis [25].

### Spectra analysis

The reproducibility of spectra was evaluated by analyzing ten *Am*. *variegatum* specimens from Kollé per sample preparation protocol as previously described [29]. The selected protocol was analyzed using an unsupervised statistical test classifying specimens according to MS spectra (i.e., Principal Component Analysis, PCA test, ClinProTools v2.2 software). The Composite Correlation Index (CCI) tool (MALDI-Biotyper v3.0. software, Bruker Daltonics), was used to assess spectra variations within each sample group according to protocol tested, as previously described [37]. CCI was computed using the standard settings of mass range 3000–12000 Da, resolution 4, 8 intervals and autocorrelation off. Higher the log score values (LSVs) and correlation values (expressed by mean ± standard deviation [SD]) reflect higher reproducibility of MS spectra and were used to determine the best protocol for sample preparation.

### Reference database creation

Based on the correlation of morphological and molecular results of tick identification, two to five specimens per species were used to assess MS spectra reproducibility from specimens of the same tick species, and the MS spectra specificity from specimens of distinct tick species. These analyses were performed with the average spectral profiles (MSP, Main Spectrum Profile) obtained from the four spots of each individual tick specimen using Flex analysis v.3.3 and ClinProTools 2.2 softwares (Bruker Daltonics). Tick species exhibiting reproducible and specific MS spectra were then included in-house MS spectra reference database. To upgrade the database, MSP reference spectra were created using spectra from at least 2 specimens per species of both genders by the automated function of the MALDI-Biotyper software v3.0. (Bruker Daltonics). MS spectra were created with an unbiased algorithm using information on the peak position, intensity and frequency [38]. The spectra files are available on request and transferable to any Bruker MALDI-TOF device.

### Blind tests

A blind test was performed with new tick specimens collected in Mali stored in 70% alcohol or frozen. A total of 451 MS spectra from tick legs, including 340 stored in alcohol and 111 frozen specimens were tested successively against the in-house MS reference spectra database (Database 1) and its upgraded version, which includes the 20 MS spectra from specimens of the 6 tick species collected in Mali and alcohol-preserved (Database 2). Among the 451 ticks tested 51 *Am*. *variegatum* and 23 *Rh (B) microplus* were fully engorged. Database 1 was composed of specimens of fresh or frozen arthropods (Table 2) [16,24,30,31,39]. Database 2 includes database 1 plus MS spectra of tick legs from 6 species stored in ethanol from the present study (Tables 3 and 4). The reliability of species identification was estimated using the LSVs obtained from the MALDI-Biotyper software v.3.3, which ranged from 0 to 3. These LSVs correspond to the degree of similarity between the MS reference spectra database and those submitted by blind tests. An LSV was obtained for each spectrum of the samples tested. Moreover, to decipher incoherent results obtained between morphological and MS identification, molecular identification of ticks was performed for the respective specimens.

**Table 2:** List of the arthropod species present in the MALDI-TOF MS database 1.

**Ticks**	*Amblyomma variegatum*, *Rh*. *sanguineus sl*, *Hyalomma marginatum rufipes*, *Ixodes ricinus*, *D*. *marginatus* and *D*. *reticulatus*, *Am*. *gemma*, *Am*. *cohaerens*, *Rh*. *e*. *evertsi*, *Rh*. *decoloratus*, *Rh*. *pulchellus*, *Rh*. *bergeoni*, *Rh*. *praetextatus*, *Hy*. *truncatum* and *Haemaphysalis leachi*
**Mosquitoes**	*Anopheles coluzzii* and *An*. *gambiae*, *An*. *funestus*, *An*. *ziemanni*, *An*. *arabiensis*, *An*. *wellcomei*, *An*. *rufipes*, *An*. *pharoensis*, *An*. *coustani*, *An*. *claviger*, *An*. *hyrcanus*, *An*. *maculipennis*, C*ulex quinquefasciatus*, *Cx*. *pipiens*, *Cx*. *modestus*, *Cx*. *insignis*, *Cx*. *neavei*, *Ae*. *albopictus*, *Aedes excrucians*, *Ae vexans*, *Ae*. *rusticus*, *Ae*. *dufouri*, *Ae*. *cinereus*, *Ae*. *fowleri*, *Ae*. *aegypti*, *Ae*. *caspius*, *Mansonia uniformis*, *Orthopodomyia reunionensis*, *Coquillettidia richiardii* and *Lutzia tigripes*
**Lice**	*Pediculus humanus corporis*, *Damalinia bovis*, *D*. *caprae*, *D*. *ovis*, *Haematopinus eurysternus*, *Linognatus vituli*, *L*. *africanus*
**Triatomine**	*Triatoma infestans*,
**Bedbugs**	*Cimex lectularius*
**Flea**	*Ctenocephalides felis*, *Ct*. *Ct*. *canis*, *Archaeopsylla erinacei*, *Xenopsylla cheopis* and *Stenoponia tripectinata*

Mosquito, tick, triatomine, bedbug reference spectra were obtained from legs protein extracts. Flea reference spectra were obtained from the whole body without abdomen protein extracts. Sandfly reference spectra were obtained from thorax, wings and legs protein extracts. Louse reference spectra were obtained from half of the body protein extracts. These species include field specimens or from insectary breeding, but also specimens collected from patients.

**Table 3:** Ticks collected per site and used for MALDI-TOF MS analyses.

Tick species	Bamako		Kollé		Bougoula-Hameau				Total number of specimens
	Number of specimens*	%	Number of specimens*	%	Number of specimens*	%	Number of specimens selected for MALDI-TOF MS	Number of MALDI-TOF MS specimens also selected for molecular analyses	
*Am*. *variegatum*	88 (39)	72.1	337 (182)	64.1	452 (208)	65.9	181	5	**877**
*Hy*. *truncatum*	8 (7)	6.6	164 (85)	31.2	88 (35)	12.8	122	5	**260**
*Hy*. *m*. *rufipes*	3 (0)	2.4	12 (5)	2.2	13 (3)	1.9	19	3	**28**
*H*. *spp*. ^#^	0	0.0	11 (11)	2.1	8 (8)	1.2	0.0	0	**19**
*Rh(Bo)*. *microplus*	16 (16)	13.1	2 (2)	0.4	8 (8)	1.2	26	3	**26**
*Rh*. *e*. *evertsi*	7 (2)	5.8	0	0	0	0	7	2	**7**
*Rh*. *sanguineus sl*	0	0	0	0	116 (52)	17	5+111^&^	2	**116**
**Total**	**122**	**100**	**526**	**100**	**685**	**100**	**471**	**20**	**1333**

*Females indicated in parentheses.

^**#**^Engorged specimens non-identifiable at the species level.

^**&**^ Ticks collected in August 2016

**Table 4:** Tick species selected to create a MALDI-TOF MS reference database, identified by molecular biology.

Morphological identification	Origin	Number of specimens tested	Intra-species similarity of 12S rRNA gene sequence (%)	Molecular identification by BLAST (Accession Number)	Query cover (%)	Similarity level with GenBank (%)
***Am***. ***variegatum***	Bamako, Kollé, Bougoula	5	100%	*Am*. *variegatum* (JF949801.1)	100%	100%
***Hy***. ***truncatum***	Bamako, Kollé, Bougoula	5	99–100%	*Hy*. *truncatum* (AF150031.1)	99–100%	96–97%
***Hy***. ***m***. ***rufipes***	Bamako, Kollé, Bougoula	3	100%	*Hy*. *m*. *rufipes* (KC817342.1)	100%	100%
***Rh(Bo) microplus***	Kollé, Bougoula	3	99–100%	*Rh(Bo) microplus* (DQ003008.1)	100%	99–100%
***Rh***. ***e***. ***evertsi***	Bamako	2	100%	*Rh*. *e*. *evertsi* (KU255856.1)	99%	100%
***Rh***. ***sanguineus sl***	Bougoula	2	100%	*Rh*. *sanguineus* (KC817342.1)	100%	100%
Rh(Bo) microplus*	Kollé, Bougoula, Bamako	26	100%	*Rh*. *microplus* (KY020993.1)	100%	100%

*Ticks identified by 16S rRNA gene

### Determination of tick infection status

These comparative analyses to determine the infectious status of ticks were made by ClinProTools v.2.2 software (Bruker Daltonics, Germany). Only tick leg MS spectra from species with at least five mono-infected or pathogen-free specimens were included in this analysis. The spectra of 30 specimens of *A*. *variegatum* infected by *R*. *africae* were compared to those of 12 uninfected specimens from the same species. Moreover, MS spectra of 36 uninfected specimens of *Hy*. *truncatum* were also compared with the spectra of 23 specimens of *Hy*. *truncatum* infected by *C*. *burnetii*.

## Results

### Tick collection and morphological identification

A total of 1,333 ticks were collected from the three sites including 406 engorged (Fig 1). A total of 1,217 were found on 44 bovine specimens and 116 on 9 dogs. Nineteen engorged females of the *Hyalomma* genus (1.55% of ticks collected) were not morphologically identified to the species level. Morphologically, six distinct tick species belonging to three genera were identified among ticks collected in September 2015 (Table 3). *Am*. *variegatum* (n = 877, 71.79%) was the overall predominant tick species collected from different sites, followed by two species of the *Hyalomma* genus, *Hy*. *truncatum* (n = 260, 21.27%) and *Hy*. *m*. *rufipes* (n = 28, 2.29%). The three other tick species, *Rh*. *(Bo*.*) microplus*, *Rh*. *e*. *evertsi* and *Rh*. *sanguineus sensus lato*, represented less than 3.10% (n = 38). The five *Rh*. *sanguineus sl* [40] specimens were all collected on a dog. All 111 ticks collected in August 2016 were identified as *Rh*. *sanguineus sl*. Three hundred sixteen of the 1,222 ticks collected from three sites in 2015 and 111 ticks in 2016 had specimens of six species randomly selected for molecular and proteomic analyses (Table 3).

### Validation of morphological identification by molecular tools on a subgroup of ticks

A total of 20 specimens, including 2 to 5 specimens per species and all specimens of *Rh (Bo.) microplus*, were randomly selected for molecular analysis. A GenBank query revealed that 12S gene sequences were available for the 6 tick species. BLAST analysis indicated high identity (i.e., a range from 99% to 100%) of 12S rRNA gene sequences among specimens classified per species according to morphological identification (Table 4). BLAST analysis revealed that these 6 tick species and all specimen of *Rh (B) microplus* had high sequence identity with their respective homolog species available in GenBank (i.e., range 96.5% to 100%; Table 4).

### Detection of bacteria in ticks

Among the ticks tested, 41.8% (197/471) were negative for the six bacteria tested, 37.4% (176/471) were positive for one bacterium and 20.8% (98/471) were found co-infected by two or three of the screened bacteria. Among the 274 specimens found positive for at least one bacteria tested, 76.6% (210/274) were infected by *Rickettsia* spp., among which *R*. *africae* was found in 87.6% (184/210) (Table 4). The amplification of the *ompA* fragment in the remaining ticks positive for *Rickettsia* spp. and negative for *R*. *africae* (n = 26) was used for identification of these *Rickettsia* spp. *R*. *aeschlimannii* and *R*. *mongolitimonae* were detected in 24 and 2 tick specimens, respectively (Table 5). Screening of all ticks for *Coxiella burnetii* revealed that 37.6% (103/274) of the specimens were positive (Table 4). Fifty-seven ticks, 20.8% (57/274) were positive in qPCR targeting the 23S rRNA of *Anaplasmataceae*. Among them, 23S rRNA amplification and sequencing was successful for 50 samples. The BLAST found broad agreement that 43 ticks were positive for *E*. *ruminantium* (GenBank accession number NR 077002.1), 2 ticks were positive for *Ehrlichia sp*. *urmitei* TCI148 (GenBank ACCN KT 364334.1) and 1 tick for *Ehrlichia sp*. *rustica* TCI141 (GenBank ACCN KT 364330.1). *A*. *marginale* was detected in 3 ticks and *A*. *sp*. *ivoriensis* TCI50 (GenBank ACCN KT 364336.1) in 1 specimen (Table 5). *Borrelia* spp. was detected in 1.1% (3/274) of ticks by qPCR. However, all standard PCR for determination of *Borrelia* species failed. No *Bartonella* spp. was detected in the ticks tested.

**Table 5:** Percentage of positive ticks detected by PCR.

Bacterium% (positives/tested)	*Am*. *variegatum*	*Hy*. *truncatum*	*Hy*. *m*. *rufipes*	*Rh (Bo) microplus*	*Rh*. *e evertsi*	*Rh*. *sanguineus sl*	Total
***Rickettsia***. ***Spp***.	**92.2% (168/181)**	**20.5% (25/122)**	**68.4% (13/19)**	**7.7% (2/26)**	**14.3% (1/7)**	**0.8% (1/116)**	**76.6% (210/274)**
***R***. ***africae***	92.2% (168/181)	9.8% (12/122)	5.2% (1/19)	7.7% (2/26)	14.3% (1/7)	-	87.6% (184/210)
***R***. ***aeschlimannii***	-	10.6% (13/122)	52.6% (10/19)	-	-	0.8% (1/116)	11.4% (24/210)
***R***. ***mongolitimonae***	-	1.6% (2/122)	-	-	-	-	1% (2/210)
***Anaplasmataceae spp***.	**8.3%(15/181)**	**10.6%(14/122)**	**10.5%(2/19)**	**80.7%(24/26)**	**42.8%(3/7)**		**20.8% (57/274)**
***A***. ***marginale***	-	-	-	11.5% (3/26)	-		6% (3/50)
***A***. ***candidatus ivoriensis***	-	-	-	0.5% (1/26)	-	-	2% (1/50)
***E***. ***ruminantium***	8.3% (15/181)	2.4% (13/122)	10.5%(2/19)	42.3% (11/26)	28.5% (2/7)	-	86% (43/50)
***Candidatus Ehrlichia rustica***	-	0.8% (1/122)	-	-	-	-	2% (1/50)
***Candidatus Ehrlichia urmitei***				1.1% (2/181)			4% (2/50)
***C***. ***burnetii***	**21.5% (39/181)**	**31.1% (38/122)**	**26.3% (5/19)**	**53.8% (14/26)**	**42.8% (3/7)**	**3.4% (4/116)**	**37.6% (103/274)**
***Borrelia spp***.	**1.1% (2/181)**	**0.8% (1/122)**	**-**	**-**	**-**	**-**	**1.1 (3/274)**

### Sample preparation protocol optimization for MALDI-TOF MS tick species identification of specimens preserved in ethanol

**Fig 3:**
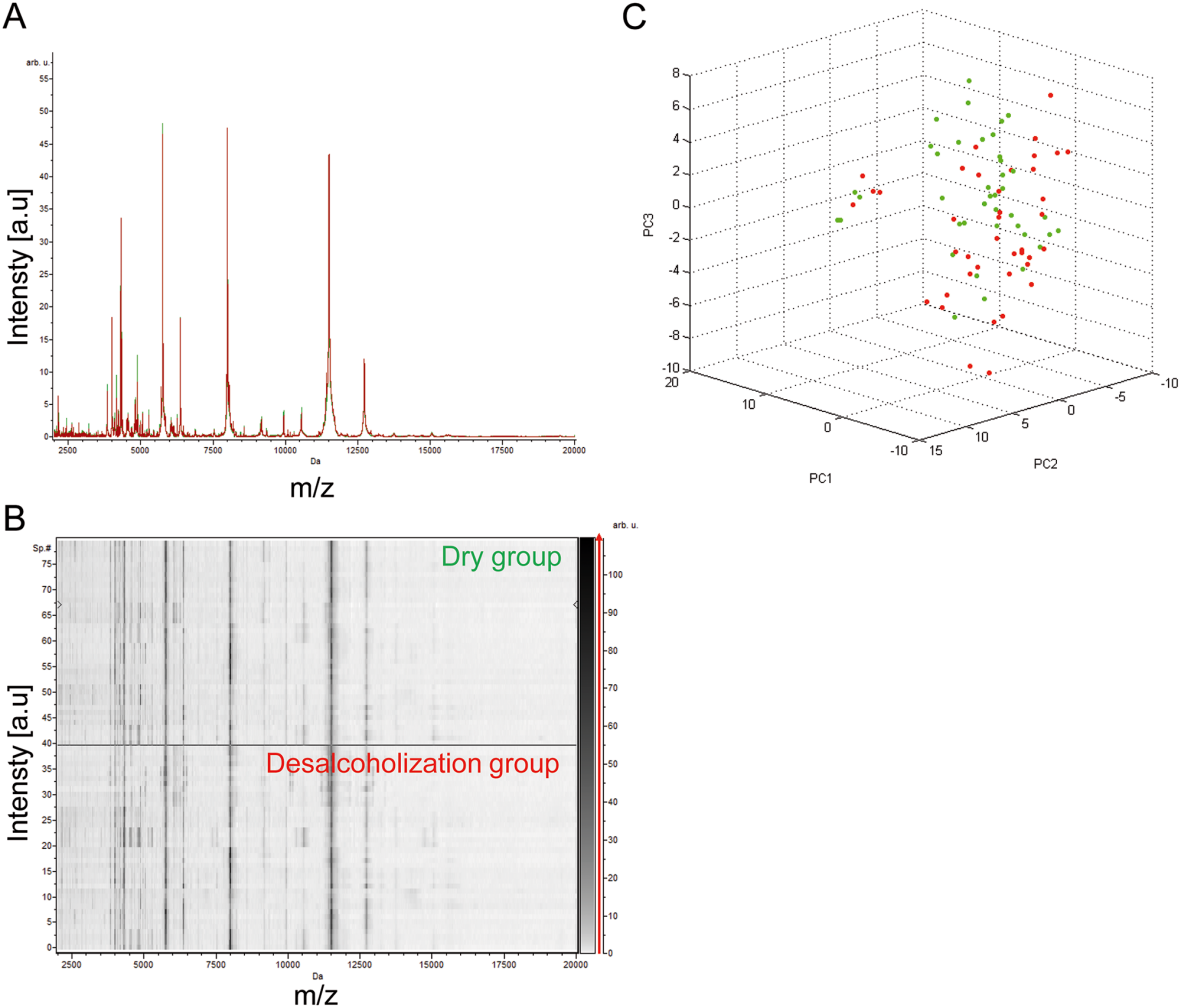
Comparison of MALDI-TOF MS spectra from legs of same tick between “de-alcoholization” and “dry” protocols. Representation of MS profiles by the superimposition of average MS profiles from legs of ticks treated by “de-alcoholization” and “dry” protocols (A), or the gel view tool (B). Tick legs MS spectra from “dry” (green dots) and “de-alcoholization” (red dots) protocols were compared by Principal Component Analysis (C); a.u., arbitrary units; m/z, mass-to-charge ratio.

**Fig 4:**
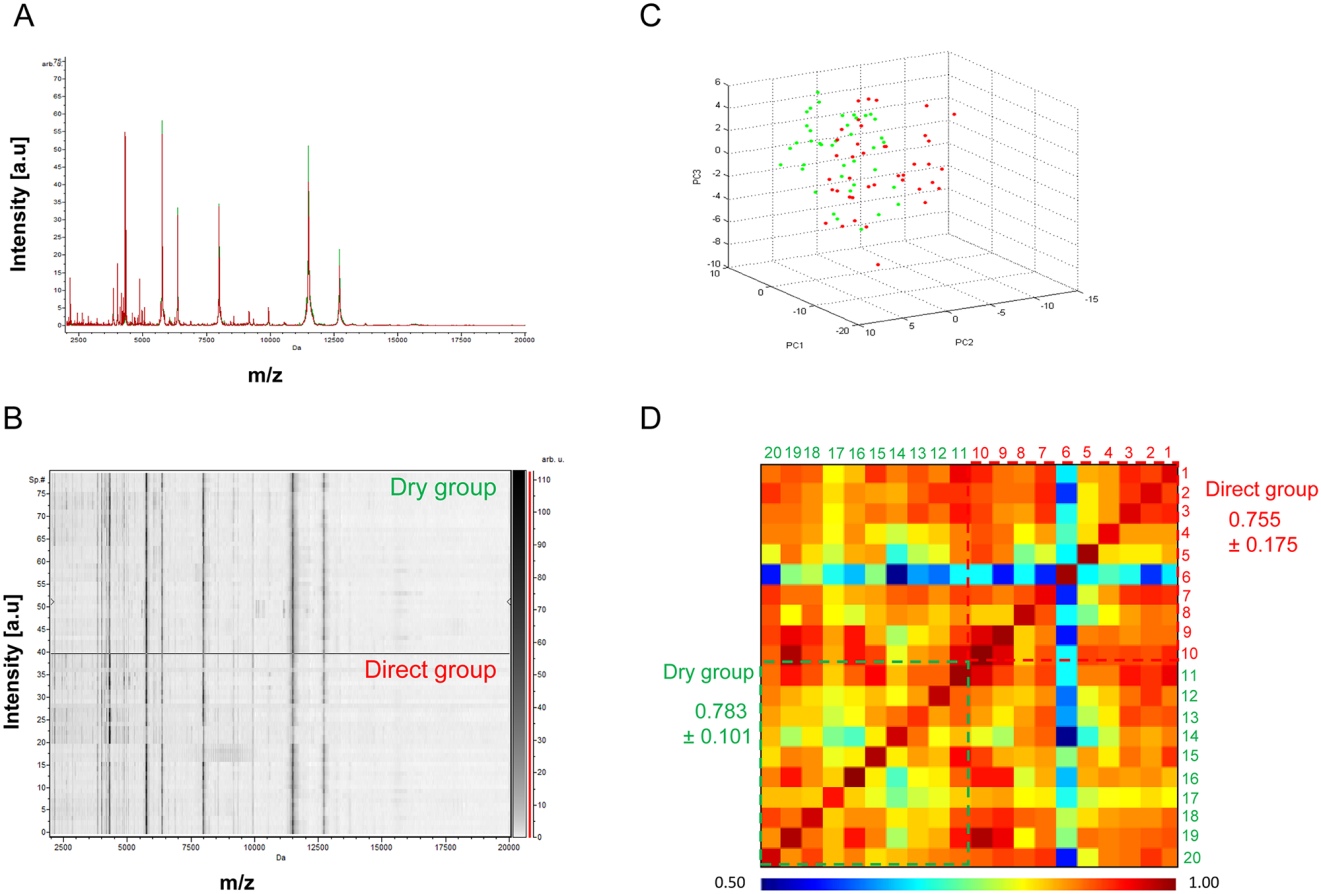
Comparison of MALDI-TOF MS spectra from legs of same tick using “dry” and “direct” protocol. Representation of MS profiles by the superimposition of average MS profiles of “dry” and “direct” protocol (A), the gel view tool of “dry” and “direct” protocol (B) and comparison by Principal Component Analysis between “dry” and “direct” protocol (C). Assessment of spectra reproducibility for two protocols using composite correlation index (CCI) (D): The rainbow colours indicate the degree of similarity between pair mass spectra comparisons ranging from red (very similar) to blue (very dissimilar). The numbers 1 to 10 are tick numbers treated by “direct” protocol and 11 to 20 those treated by “dry” protocol.

A comparison of our current reference sample preparation method (i.e., “de-alcoholization”) with the “dry” and “direct” methods was performed [31]. The best method was selected on the following criteria: reproducibility and intensity of MS spectra, low handling and simplicity of the protocol. To exclude inter-individual variability, protocols were successively compared by pairs, and then the four right legs were used for one protocol and the four left legs from the same tick for the other. Then, ten specimens of both genders tested per protocol, five males and five females, were included. For all these experiments, morphologically identified ticks from Kollé (*Am*. *variegatum)* were used. The first comparison concerned the “de-alcoholization” and “dry” protocols (Fig 2A). The visual comparison of MS profiles between these two groups using the gel view tool and the superimposition of average MS profiles in each condition using ClinProTools software (Bruker) did not reveal differences in peak position between the two protocols (Fig 3A and 3B). This reproducibility of the profiles was analyzed using an unsupervised statistical test classifying specimens according to MS spectra (i.e., Principal Component Analysis, PCA test, ClinProTools software). The mixing of both groups on the graphical representation confirmed the absence of differences between both groups (Fig 3C). Thus, the “dry” protocol was preferred compared to the “de-alcoholization” protocol, the latter considered to be more time-consuming and fastidious. The second comparison concerned the “dry” and “direct” protocols, using ten *Am*. *variegatum* specimens from both genders (Fig 2B). The comparison of MS profiles between these two groups, either by gel view, superimposition or PCA (Fig 4A, 4B and 4C), could not determine the more relevant method. The Composite Correlation Index (CCI) tool revealed a higher CCI (LSV mean±SD: 0.783±0.101; Fig 4D) for the “dry” protocol compared to “direct” (LSV mean±SD: 0.755±0.175; Fig 4D). These results were in agreement with the gel view showing a higher visual homogeneity of the MS spectra from the “dry” group. Finally, the “dry” protocol appeared consistently to be the more reproducible and low-handling procedure for the preparation of ethanol-stored ticks for MS analysis, and was chosen for the next experiments of the present study.

### Intra-species reproducibility and inter-species specificity of MS spectra

**Fig 5:**
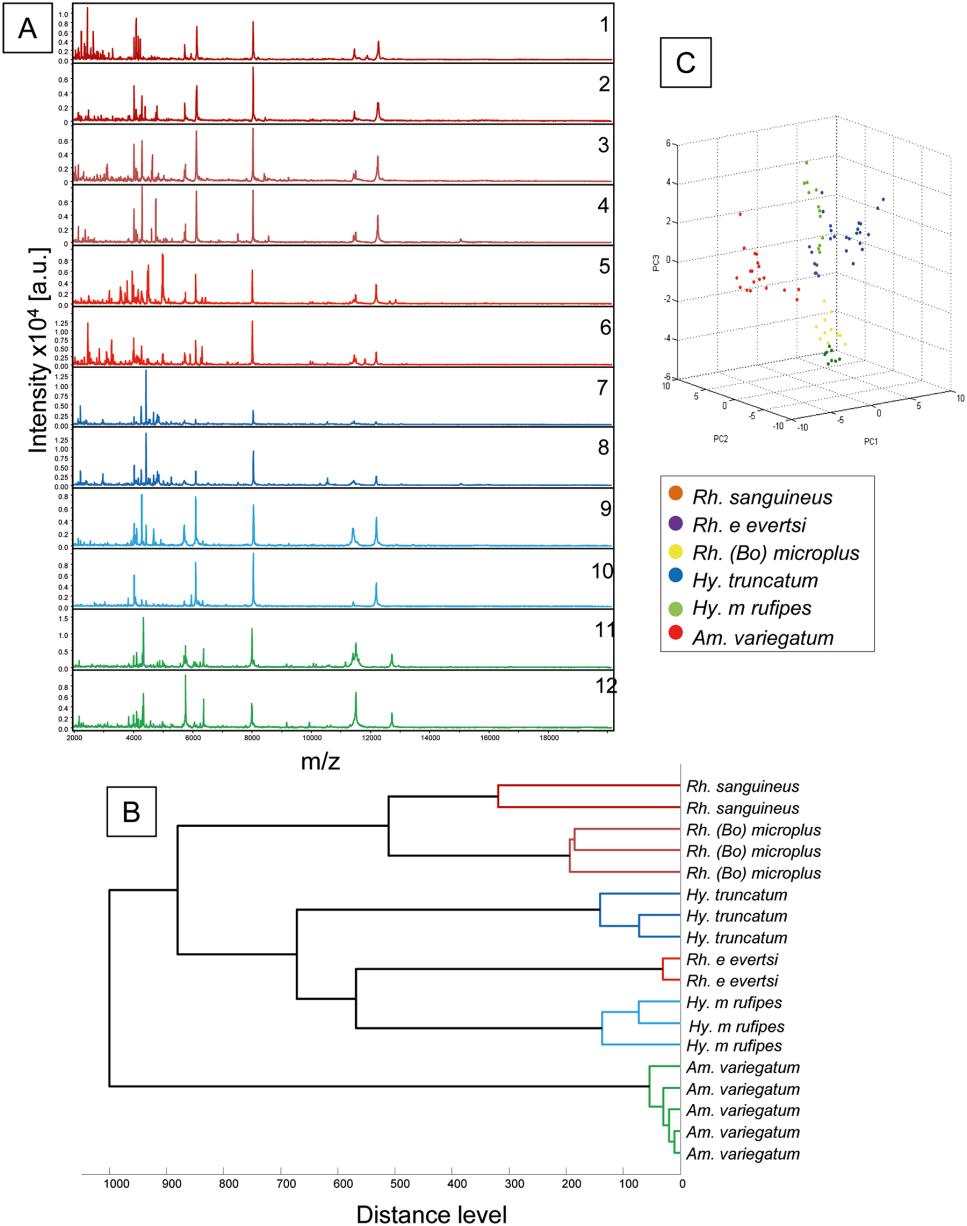
Specific MALDI-TOF MS spectra of six species of ticks using for database creation. (A) Representation of leg MS spectra from *Rh*. *sanguineus sl* (1, 2), *Rh*. *(B) microplus* (3, 4) *Rh*. *e*. *evertsi* (5, 6), *Hy*. *truncatum* (7, 8), *Hy*. *m*. *rufipes* 9, 10), *Am*. *variegatum* (11, 12). (B) Dendrogram constructed using 2 to 5 representative MS spectra from 6 distinct tick species. (C) Principal Component Analysis performed with 20 specimens of six tick species; a.u., arbitrary units; m/z, mass-to-charge ratio.

Twenty ticks, including several specimens per species coming from distinct localities, were identified by sequencing 12S tick gene. Their non-infected status was also controlled for the microorganisms tested in the present work by q PCR. These specimens were selected for evaluating intra-species reproducibility and inter-species specificity of MS spectra. Comparison of the MS spectra with Flex analysis software indicated reproducibility of the MS profiles between tick specimens from the same species (Fig 5A). Moreover, the visual comparison of MS profiles indicated a clear distinction of spectra according to species. To reinforce the specificity of MS profiles according to tick species, MS profiles from these 20 specimens were used to generate a dendrogram and PCA (Fig 5B and 5C). Clustering analysis revealed a gathering on distinct branches of ticks according to species. However, at the genus level, all specimens from the *Rhipicephalus* genus were not clustered in the same part of the dendrogram. The profile of the spectra of specimens preserved in alcohol was different from those of fresh specimens of the same species; this difference was also observed between manual sample homogenization and automated sample crushing using the TissueLyser apparatus.

### MS reference spectra database upgrading and blind test for tick identification

To assess the efficacy of the in-house MS reference spectra database, named database 1 (DB 1) to correctly identify tick specimens preserved in alcohol, half of the MS spectra from ticks included in the present study were randomly selected. Then, MS spectra from 178 specimens including 60 *Am*. *variegatum*, 64 *Hy*. *truncatum*, 16 *Hy*. *m*. *rufipes*, 26 *Rh*. *(Bo*.*) microplus*, 7 *Rh*. *e*. *evertsi* and 5 *Rh*. *sanguineus sl* were queried against the DB 1 spectra database. The blind test against DB 1 revealed correct identification for some specimens of *Hy*. *truncatum* (n = 5) and *Hy*. *m*. *rufipes* (n = 5), with LSVs > 1.8 (Table 6). For the remaining ticks (n = 168), all LSVs were ˂ 1.8 [24]. Tick MS spectra from 20 specimens, including 6 species identified morphologically and molecularly in this work, were added to DB 1, which was then renamed DB 2 (Table 4). Thereafter, the leg spectra of the 451 morphologically-identified ticks, including 340 stored in alcohol and 111 frozen, were queried against DB 2. Among the 451 ticks tested 51 *Am*. *variegatum* and 23 *Rh (Bo.) microplus* were fully engorged.

**Table 6:** The number of ticks used to perform the blind test and percentage of correct identification.

Species	Number of specimens used for BT1	High LSVs obtained from BT1 against DB1^¤^	Number of concordant ID between morphology and MS^#^	Number of specimens added to DB1	Number of specimens used for BT2	High LSVs obtained from BT2 against DB2^¤^	Tick species ID by MS	Molecular ID^$^	Concordance of MS ID with morphology and molecular (%)^&^
*Am*. *variegatum*	60	*[1*.*10–1*.*73] (n = 60)*	n.d.	5	177	[1.81–2.63] (n = 173)	*Am*. *variegatum*	/	100
						[2.06–2.23] (n = 4)	*Hy*. *truncatum*	*Hy*. *truncatum*	
*Hy*. *truncatum*	64	[1.81–1.95] (n = 7)	5	5	117	[1.90–2.72] (n = 109)	*Hy*. *truncatum*	/	98.3
		*[1*.*37–1*.*79] (n = 57)*	n.d.			[1.98–2.35] (n = 5)	*Hy*. *m*. *rufipes*	*Hy*. *truncatum*	
						[1.87–2.35] (n = 3)	*Am*. *Variegatum*	*Am*. *Variegatum*	
*Hy*. *m*. *rufipes*	16	[1.81–1.91] (n = 5)	5	3	15	[2.10–2.77] (n = 12)	*Hy*. *m*. *rufipes*	/	100
		*[1*.*54–1*.*79] (n = 11)*	n.d.			[2.10–2.32] (n = 3)	*Hy*. *truncatum*	*Hy*. *truncatum*	
*Rh (Bo) microplus* ^€^	26	*[1*.*20–1*.*72] (n = 26)*	n.d.	3	23	[1.91–2.71] (n = 23)	*Rh (Bo) microplus*	/	100
*Rh*. *e*. *evertsi*	7	*[1*.*51–1*.*61] (n = 7)*	n.d.	2	5	[1.90–2.49] (n = 5)	*Rh*. *e*. *evertsi*	/	100
*Rh*. *sanguineus sl*	5	*[1*.*20–1*.*48] (n = 5)*	n.d.	2	3	[1.89–2.10] (n = 3)	*Rh*. *sanguineus sl*	/	100
*Rh*. *sanguineus sl*	/	/	/		111	[1.90–2.29]	*Rh*. *sanguineus sl*	/	100
**Total**	**178**				**451**				**99.6**

^€^Tick species stored in alcohol not included in the DB1 [31]

*Tick specimens stored frozen.

^¤^ The number of specimens included in each range of LSVs (above and *below* 1.8) are in parentheses.

^$^Molecular biology of tick species ID done only on discordant MS and morphological results.

^&^Percentages of tick species with ID concordance between MS and morphological results plus molecular determination.

BT, blind test; DB, database; ID, identification; LSVs, log score values; MS, mass spectrometry; n.d., not determined.

The results of this second interrogation (blind test 2, BT2) showed 96.7% (325/340) concordance between morphological identification and MALDI-TOF MS identification. The percentage of concordant identification with morphology was 100% for *Rh (Bo) microplus*, *Rh*. *e*. *evertsi* and *Rh*. *sanguineus sl* stored in alcohol, with LSVs ranging from 1.89 to 2.71 (Table 6). A total of 15 specimens presented divergent identification between morphological and MALDI-TOF MS identification. To eliminate any doubt, these 15 specimens were submitted to molecular identification. Sequencing of the 12S gene confirmed the identification obtained by MALDI-TOF MS for 13 specimens (Table 6). The remaining 2 specimens identified as *Hy*. *m*. *rufipes* by MALDI-TOF MS were finally classified as *Hy*. *truncatum* by molecular biology, confirming morphological identification. All fully engorged ticks were correctly identified by MALDI-TOF MS. The percentage of correct MALDI-TOF MS identification for all species was 99.6% (449/451) (Table 6).

### Determination of tick bacterial infectious status by MALDI-TOF MS

The comparison of MS profiles between 30 *Am*. *variegatum* uninfected and 12 infected by *R*. *africae* using the gel view tool and Principal Component Analysis by ClinProTools software (Bruker), revealed no differences between the two groups (S1 Fig). The same observation was made by comparing of MS profiles of 36 *Hy*. *truncatum* uninfected and 23 *Hy*. *truncatum* infected by *C*. *burnetii* (S1C and S1D Fig).

## Discussion

MALDI-TOF MS has revolutionized clinical microbiology by its use in the routine identification of bacteria [41,42] and archaea [43]. Even if the MALDI-TOF MS device acquisition could be expensive, its use for entomological analyzes induces low additional costs because reagents used for this high-throughput technique are economical and data analyses are simple and rapid compared to morphological and molecular methods [44]. This fast, economical and accurate proteomic tool has since been applied to the identification of arthropods: culicoides biting midges [45], mosquitoes[39,46,47], phlebotomine sand flies [48,49], fleas [30] and tsetse flies [50,51]. MALDI-TOF MS has also been proposed for identifying tick species which are laboratory-reared, collected in the field or on mammalian hosts, by analyzing whole specimens [22] or legs only [23,24]. More recently, preliminary studies have investigated the capacity of MALDI-TOF MS to differentiate ticks infected or not by *Borrelia spp*. or spotted fever group rickettsiae [25–27], and to detect the *Plasmodium* in anopheles [44]. However, tick collection is usually far from the analytical laboratories, requiring proper storage of samples. Although the alcohol storage mode is cheaper and easier, especially in African countries, previous studies reported that the use of fresh (i.e., recently dead) or frozen specimens led to more reproducible and better MS spectra, compared to the alcohol storage mode for ticks [24,28], and also for other arthropod families [29,30]. Recently, the application of MALDI-TOF MS for identification of ticks collected in the field in East Africa and preserved in alcohol has allowed reliable identification [23]. More recently, the discriminatory power of MALDI MS-TOF for the correct identification of ixodid tick specimens collected in the field in Ethiopia, which were preserved in 70% ethanol for about two years, was reported [31].

In this study, the morphological identification of ticks revealed the presence of six species, including *Am*. *variegatum*, *Hy*. *truncatum*, *Hy*. *m*. *rufipes*, *Rh*. *(Bo) microplus* and *Rh*. *e*. *evertsi* that were collected from cattle and *Rh*. *sanguineus sl* from dogs. *Rh*. *e*. *evertsi* was found only in Bamako, while all other species of ticks were found on cattle in the three locations. In support of these morphological identification results, several studies have reported the presence of these tick species in Mali, except for *Rh*. *(Bo) microplus* [7–9]. *Rh*. *(Bo) microplus*, which is a southeast Asian tick, was introduced in the southeast of Africa (South Africa, Zambia, Tanzania and Malawi) by cattle from Madagascar [9]. It was reported in West Africa (Ivory Coast) for the first time in 2007 [52]. The presence of *Rh*. *(Bo) microplus* has only been found in three other countries of West Africa (Mali, Benin and Burkina Faso) [53]. Biguezoton et al (2016) and Boka et al (2017) found that *Rh (B) microplus* represent 70% and 63.2% of ticks in Burkina Faso and Benin and Ivory Coast respectively [54, 55]. Our study confirms the presence of this species in 3 localities in Mali, which could indicate its rapid spread and its probable installation in Mali. As expected, *Am*. *variegatum* was the most prevalent species in the three sites of the present study [10].

To confirm the morphological identification of tick specimens that were used for creating the MALDI-TOF MS database, sequencing of the 12S rRNA gene was performed. The 12S rRNA gene was chosen to validate identification because this gene is known as a reliable tool for molecular identification of ixodid ticks [16]. The coverage percentages and identity between the sequences of specimens of the same species were from 99 to 100% for all species of ticks. Percentages of identity and coverage of sequences *Am*. *variegatum*, *Hy*. *m*. *rufipes*, *Rh*. *(Bo) microplus*, *Rh*. *e*. *evertsi* and *Rh*. *sanguineus sl were* 99–100% with sequences of the same species available in GenBank. Interestingly, lower sequence identities (96–97%) of *Hy*. *truncatum* compared to the corresponding reference sequence in GenBank were observed. It could be hypothesized that the sequence differences could correspond to genetic variation within ticks of the same species adapted to different geographic regions of a country or countries, as previously described [56]. The difference between the sequences of 12S rRNA genes of *Hy*. *truncatum* collected in Mali and that available on GenBank tick collected in Zimbabwe [57] could explain these genetic variations. In the future, the sequencing of a second gene target, such as 16S or COI, could be performed to further study these variations [58].

In this study, DNA from *Rickettsia spp*. was detected in 76.6% of infected ticks collected from cattle, among which *R*. *africae* was found in 87.6% (184/210). *R*. *africae* was detected in 92.2% of *Am*. *variegatum*, a cattle tick found throughout sub-Saharan Africa. Such high prevalence of *R*. *africae* in *Am*. *variegatum* has already been reported [59–61]. *R*. *africae* was also detected in *Rhipicephalus* spp. and *Hyalomma* spp., respectively 7.9% and 9.2%. Other recent studies have detected *R*. *africae* in other tick genera, including *Rhipicephalus* and *Hyalomma* [59,62,63]. *R*. *africae* is the etiological agent of African tick-bite fever in humans (ATBF) [64]. *R*. *aeschlimannii* have been observed in *Hyalomma spp*., with 9% and 52.6% respectively in *Hy*. *truncatum* and *Hy*. *m*. *rufipes*. These data are comparable with those of previous studies that reported 45% to 55% of *Hy*. *m*. *rufipes* and 6% to 7% of *Hy*. *truncatum* were DNA carriers of *R*. *aeschlimannii* in Senegal [63], and 44% and 11% in Ivory Coast [59]. The sequences of *R*. *aeschlimannii* identified in our work were identical to those of *R*. *aeschlimannii*, previously detected in *Hy*. *truncatum* collected in Senegal (GenBank accession number HM050276.1). *R*. *aeschlimannii* is an agent of spotted fever in humans [64]. *R*. *aeschlimannii* is found in sub-Saharan Africa, North Africa, Europe and Asia [11,65]. Our results confirm a large prevalence of this pathogen in Mali.

For the first time, the presence of *R*. *mongolitimonae* was identified in *Hy*. *truncatum* from Mali. It had been previously detected in *Hy*. *truncatum* from the countries bordering Mali, including Niger [11] and Senegal [63]. *R*. *mongolitimonae* 12S sequence of the present study were 99% identical with the same sequence fragment of a strain previously isolated from a patient from Algeria (GenBank DQ097081.1).

Until now, two *Borrelia* species have been identified in Mali, *B*. *crocidurae* in the soft tick (*O*. *sonrai*) and *B*. *theileri* in the hard tick (*Rh*. *geigyi*) [12,13]. Our results show the presence of *Borrelia* spp. in 2 specimen of *Am*. *variegatum* and 1 of *Hy*. *truncatum* by qPCR using 16S *Borrelia* and ITS4 genes. Similarly, Ehounoud et al. previously reported the presence of *Borrelia spp*. in the same tick species in Ivory Coast [59]. Unfortunately, no PCR products using standard amplification were obtained for any of these ticks. This failing could be explained by the higher sensitivity of qPCR compared to standard PCR [66].

In the present work, *C*. *burnetii*, the agent of Q fever, was detected for the first time in ticks in Mali, with a prevalence of 33.4% in the six tick species identified. These results differ from those of Ehounoud et al. in Ivory Coast, who found only one tick infected with *C*. *burnetii* [59]. Q fever is a ubiquitous zoonotic disease caused by *C*. *burnetii*. It is poorly documented in Africa. A recent study conducted in febrile African patients found one male adult patient (0.3%) infected with *C*. *burnetii* in Algeria and six patients (0.5%) in Senegal [67]. However, in another study conducted in Senegal, *C*. *burnetii* was detected in humans as well as in ticks [68].

The *Anaplasmataceae* bacteria family was previously considered to be pathogens of veterinary importance [59]. However, in recent decades, many agents of this family have been described in humans [69]. Here, we reveal the presence of *A*. *marginale* in 11.5% of *Rh (Bo*.*) microplus*. This is the first demonstration of the presence of *A*. *marginale*, the agent of bovine anaplasmosis [70] in Mali. *A*. *marginale* is an intracellular bacterium responsible for bovine anaplasmosis which manifests with anemia and jaundice [64]. Also, *E*. *ruminantium* was found in *Am*. *variegatum*, *Hy*. *truncatum*, *Rh (Bo*.*) microplus*, and *Rh*. *e*. *evertsi*. The prevalence of *E*. *ruminantium* was 13.9% in ticks. Potential new species of *Ehrlichia* and *Anaplasma* (*E*. *sp urmitei* TCI148, *E*. *sp rustica* TCI141 and *A*. *sp ivoriensis* TCI50) have been detected in *Rh (Bo*.*) microplus* and *Hy*. *truncatum*. These bacteria had already been detected in ticks from Ivory Coast [59].

However, co-infections have been found in the ticks in this study. The percentage of co-infected ticks was 23.1% (109/471), and we describe for the first time multiple co-infections in ticks in Mali. Recently, multiple co-infections in ticks have been reported in Ivory Coast; these co-infections systematically involved *R*. *africae* [59]. The percentage of ticks co-infected was higher in our study than that obtained in Ivory Coast [59].

To avoid bias, we choose to query the MS spectra of 178 specimens of ticks, including 6 species against DB 1 which includes several families of arthropods, including mosquitoes. We constantly improve it with new specimens collected in the field and find it more relevant to carry out a total interrogation without the knowledge without any filter on a specific family. The results of the blind test revealed correct identification in 10 specimens only with high log score values, even though this database contained the same tick species that were also preserved in alcohol. This misidentification could be attributed to several factors: (i) the method used for sample crushing (initially manually, and here an automatic apparatus was used as previously described [28], (ii) the difference in storage time (6 months here vs 3 years in the previous study), (iii) the geographical distance (Mali vs. Ethiopia), which could have consequences on MS spectra profiles, as observed also at the genetic level. This last phenomenon had already been reported in other studies of sand flies [71], mosquito immature stages [46] and ticks [31]. Conversely, when database 1 was upgraded with 20 spectra of the six tick species of our study, the blind test of all ticks revealed 95.60% (325/340) correct identification for tick species stored in alcohol. However, the remaining fifteen ticks (4.40%) with inconsistent identification between morphological and MALDI-TOF MS tools were subjected to molecular biology to determine the real identification of these specimens. The molecular biology results confirmed those of MALDI-TOF MS for 13 of these specimens. Two *Hy*. *truncatum* specimens were misidentified by MALDI-TOF MS. The reasons for the misidentification of the two specimens remain unknown. Additionally, all ticks frozenly stored were correctly identified by the blind test.

The results of this work show that MALDI-TOF MS is superior to morphological identification, as the correct identification percentage is 99.6% for all tested. It is also interesting because there are fewer entomologists able to identify ticks and the morphological identification keys are not always available. Another advantage of MALDI-TOF is that it can identify ticks that are completely engorged or damaged, for which morphological characteristics can be deformed or even disappear making morphological identification difficult or impossible. Conversely, the proteomic strategy proposed here, does not require specific skill or expertise, reagents are very cheap so the running cost is very low compared to a molecular biology. The current limiting factors of MALDI-TOF MS analysis are the small diversity of tick species included in the MS spectra reference database and the relative elevate cost to acquire the machine. Nevertheless, it high-throughput and large application for microorganisms identification either in research or medical diagnosis, do of this emerging tool a highly competitive method also for medical entomology studies. It is likely that MALDI-TOF MS will realize similar revolution in medical entomology as it was occurred in microbiology.

Our results confirm those of previous studies, according to which MALDI-TOF MS could be used for identification of ticks preserved in alcohol, but it requires the creation of a database with specimens stored in the same condition [31].

In our work, MALDI-TOF MS analysis was not able to differentiate ticks which were infected or not by the bacteria that were screened. However, preliminary studies from our laboratory seemed promising, as MALDI TOF analysis allowed differentiation of ticks infected or not by *Borrelia spp*. or spotted fever group rickettsiae [26,27]. The failing of bacteria-pathogen detection by MALDI-TOF MS could be attributed to several factors. The storage mode, fresh versus alcohol, might play a role. Moreover, the infectious status of these ticks was controlled against some bacteria pathogens, however, it was possible that they were infected by others pathogens not researched in the present study, which could impaired the determination of specific MS profiles for each associated pathogens. These factors could alter MS spectra profiles between uninfected and infected ticks. More studies are needed to explore the capacities of MALDI TOF to detect tick infectious status.

To conclude, the present work has confirmed that MALDI-TOF MS may represent a rapid and inexpensive alternative tool for accurate identification of ticks collected in the field and stored in alcohol. The recent demonstration of the use of MALDI-TOF MS for identification of ticks and associated pathogens requires further investigation.

## Supporting information

S1 Fig Comparison of MALDI-TOF MS spectra of ticks uninfected and infected. Representation of MS profiles of *Am*. *variegatum* unifected (Red) and infected by *R*. *africae* (Green) (A, B) and *Hy*.*truncatun* unifected (Red), infected by *C*.*burnetii* (Green) (C, D).
(TIF)
