# Expression of Concern: Molecular and MALDI-TOF identification of ticks and tick-associated bacteria in Mali

**DOI:** 10.1371/journal.pntd.0010976

**Published:** 2022-12-13

**Authors:** The PLOS Neglected Tropical Diseases Editors

This article [1] has been identified as one of a series of submissions for which we have concerns about the reported research ethics approval information and the article’s adherence to PLOS research ethics policies.

PLOS will be investigating these concerns in accordance with COPE guidance and journal policies. Meanwhile, the *PLOS Neglected Tropical Diseases* Editors issue this Expression of Concern.
