# Correction: Molecular and MALDI-TOF identification of ticks and tick-associated bacteria in Mali

**DOI:** 10.1371/journal.pntd.0014311

**Published:** 2026-05-12

**Authors:** The PLOS Neglected Tropical Diseases Editors

The *PLOS Neglected Tropical Diseases* Editors issue this notice to resolve the concerns underlying the previously published Expression of Concern on this article [1,2].

Following its publication, PLOS investigated this article as part of a series of articles with concerns pertaining to the reported ethical oversight and the article’s adherence to PLOS research ethics policies.

The article’s Materials and methods section reports the collection of ticks from domestic animals and cattle between September 2015 and August 2016. The Ethical considerations subsection of the Materials and methods section states that the protocols for the tick collection were cleared by the FMPOS IRB in 2015 and 2016, that verbal consent was obtained from the managers of the livestock selected for tick sampling, and that the collection of ticks did not involve national parks, other protected areas, or endangered species.

Following editorial communications, the corresponding author stated that the study was conducted in accordance with local procedures and law, and provided the documents listed below for editorial review:

- N°2016/113/CE/FMPOS issued on August 16, 2016, by the Ministry of Higher Education and Scientific Research in Mali, which grants ethics approval for a research project titled “*Enquête de prévalences, de surveillance d’investigation des viroses émergentes et ré-émergentes et des causes infectieuses de fièvre au Mali.”*
- N°2022/305/CE/USTTB issued on December 2, 2022, by the Ethics Committee of the Université des Sciences, des Techniques et des Technologies de Bamako, Mali. The document is a letter from the president of the institute’s Ethics Committee stating that no further ethics approval was required for this publication, as the removal of the ticks from domesticated animals was voluntary and identifying information of the animals’ owners was not recorded.

PLOS did not receive a copy of the 2015 FMPOS ethics approval documentation reported in the article, and the 2016 FMPOS approval does not provide prospective approval for the full sample collection period. However, in light of the documentation received from the Ethics Committee of the Université des Sciences, des Techniques et des Technologies, Mali, confirming that no further ethics approval would have been required for the study, the *PLOS Neglected Tropical Diseases* Editors consider the ethics approval concerns resolved.

This Correction supersedes the prior Expression of Concern [2].

## References

[1] Diarra AZ, Almeras L, Laroche M, Berenger J-M, Koné AK, Bocoum Z, et al. Molecular and MALDI-TOF identification of ticks and tick-associated bacteria in Mali. PLoS Negl Trop Dis. 2017;11(7):e0005762. doi: 10.1371/journal.pntd.0005762 28742123 PMC5542699

[2] The *PLOS Neglected Tropical Diseases* Editors. Expression of Concern: Molecular and MALDI-TOF identification of ticks and tick-associated bacteria in Mali. PLoS Negl Trop Dis. 2022;16(12):e0010976. doi: 10.1371/journal.pntd.0010976 36512525 PMC9747001

