# Supplementary figures and images for: Molecular and MALDI-TOF identification of ticks and tick-associated bacteria in Mali
Source: PLoS Negl Trop Dis. 2017 Jul 24;11(7):e0005762. doi: 10.1371/journal.pntd.0005762 (PMC5542699; doi:10.1371/journal.pntd.0005762)

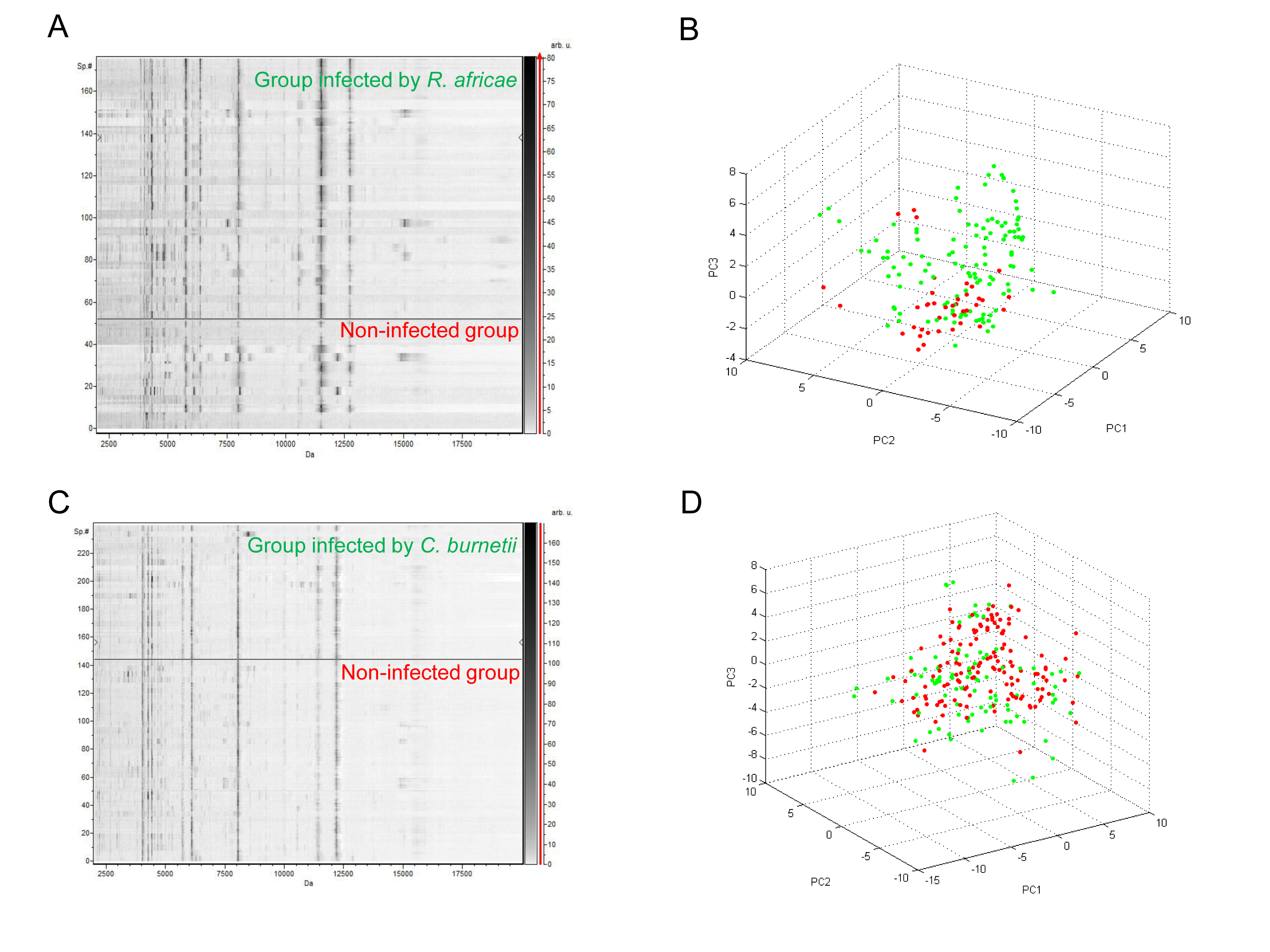

Supplement: S1 Fig — Representation of MS profiles of Am. variegatum unifected (Red) and infected by R. africae (Green) (A, B) and Hy.truncatun unifected (Red), infected by C.burnetii (Green) (C, D). (TIF) [file pntd.0005762.s001.tif]
